# Mercury poisoning in a patient with thrombotic events: A case report

**DOI:** 10.1016/j.radcr.2025.10.033

**Published:** 2025-11-11

**Authors:** Alejandra Guarnizo-Lozano, Sara Saltarín-Leyton, Mayra Murillo, Felipe Aluja-Jaramillo, Oscar Sanabria

**Affiliations:** a Departamento de Medicina Interna, Hospital Universitario San Ignacio, Bogotá, Colombia; b Departamento de Medicina Interna, Pontificia Universidad Javeriana, Bogotá, Colombia; c Departamento de Radiología e imágenes diagnósticas, Hospital Universitario San Ignacio, Bogotá, Colombia; d Facultad de Medicina, Pontificia Universidad Javeriana, Bogotá, Colombia

**Keywords:** Mercury poisoning, Thrombophilia, Multidetector computer tomography, Case report

## Abstract

Mercury poisoning is an underdiagnosed condition in our setting. It affects multiple organs and systems, often presenting with variable and nonspecific symptoms that may mimic more common diseases. We report the case of a 42-year-old female patient who presented with multiple arterial and venous thrombotic events, cardiovascular complications, progressive cognitive impairment, and long-standing renal dysfunction. Imaging studies revealed embolic phenomena characterized by hyperdense material suggestive of metallic emboli in the thorax, abdomen, and pelvis, raising suspicion of heavy metal poisoning. Diagnosis was confirmed by a 24-hour urinary mercury concentration of 64.03 µg/L. Chelation therapy with the available agent in our setting was initiated, along with intravenous N-acetylcysteine. Selenium was not administered due to its unavailability. Mercury intoxication poses a diagnostic challenge due to its nonspecific clinical manifestations, which may be attributed to more prevalent pathologies in the region.

## Introduction

Mercury is a chemical element found in various forms—elemental mercury, inorganic mercury, and methylmercury [1]. One of the most common sources of exposure is the release of elemental mercury vapor into the atmosphere, which may subsequently contaminate soil and water sources [1]. In humans, the most frequent routes of exposure are oral, via ingestion of contaminated water or mercury-laden fish and inhalation of elemental mercury vapor [2]. Intravenous exposure is rare but has been documented in case reports [3,4]. Pulmonary embolism due to metallic material may occur in cases of intravenous administration [5].

The physiological effects of mercury depend on the route of exposure, dosage, duration of exposure, and its chemical form. The biological half-life is variable and may reach up to 70 days, with primary excretion via feces [1]. Mercury deposits can be found in several organs including lungs, liver, kidneys, skeletal and cardiac muscle, and glandular tissues such as breast, thyroid, pancreas, and prostate [6].

Clinical manifestations of mercury toxicity may initially include nonspecific and gastrointestinal symptoms such as nausea, vomiting, and abdominal pain, due to its corrosive effects. Chronic exposure may lead to multi-organ involvement, notably renal dysfunction and central nervous system compromise, manifesting as cognitive decline and mood alterations [1,7]. Oxidative stress-induced endothelial injury, platelet activation, and increased activity of coagulation factors are believed to contribute to both acute and chronic thrombotic events associated with mercury intoxication [8].

We present a case of mercury poisoning manifesting primarily with multiple thrombotic events.

## Case report

A 42-year-old woman from Bogotá presented to the emergency department with a medical history of hypertension, type 2 diabetes mellitus, morbid obesity, chronic kidney disease, a previous ischemic stroke 1 year prior, and major depressive disorder with self-injurious behavior. She reported a 1-day history of sudden-onset dyspnea, chest pain, dry cough, and bilateral lower limb edema. At admission, blood pressure 121/74 mm Hg heart rate 108 beats per minute, respiratory rate 20 breaths per minute, saturation was 60% on pulse oximetry.

On physical examination, the patient exhibited decreased consciousness, diaphoresis, central and peripheral cyanosis, and signs of respiratory distress with accessory muscle use. Pulmonary auscultation revealed crackles. She also had lower limb edema and prolonged capillary refill. Endotracheal intubation was performed for invasive mechanical ventilation, and norepinephrine was initiated due to hypoxemic respiratory failure and shock.

**Fig. 1:**
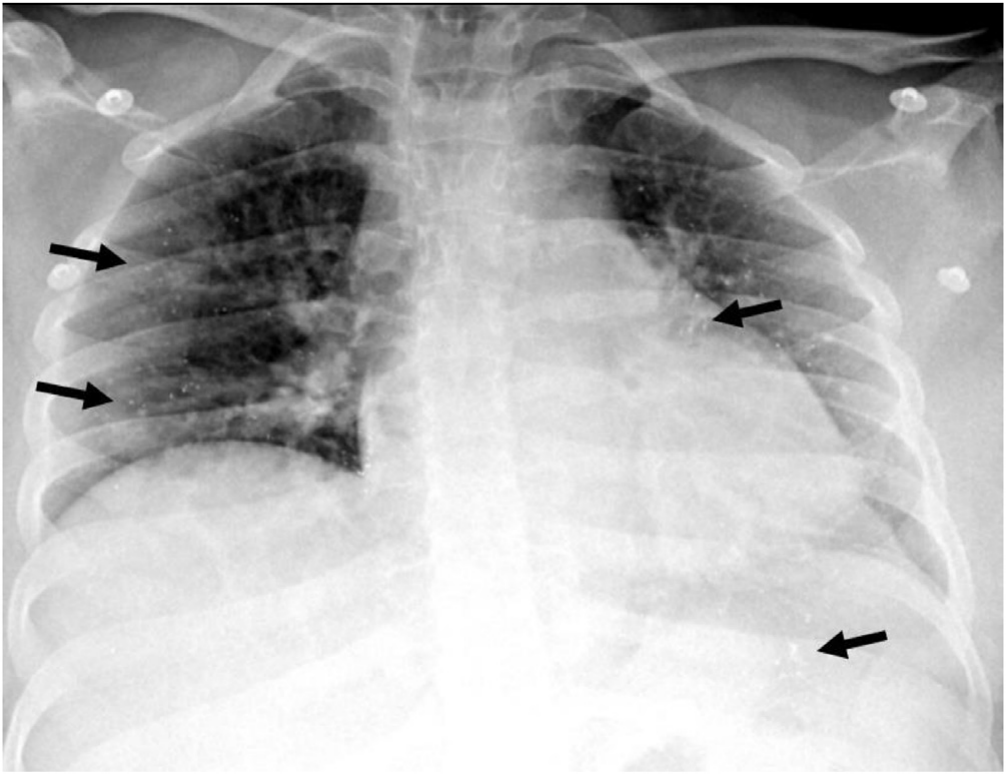
Chest radiography, AP view. There are some radiopaque nodules in the lung parenchyma. These nodules are closely related to the pulmonary vasculature.

Laboratory results showed leukocytosis (15.9 × 10⁹/L), neutrophilia (14.2 × 10⁹/L), lymphopenia (800 × 10⁹/L), hemoglobin 9 g/dL, hematocrit 31.2%, platelet count 258,000/µL, elevated troponin I (38,115 pg/mL), NT-proBNP (13,558 pg/mL), creatinine 0.54 mg/dL, and proteinuria (1.8 g/24 h). Chest radiography in AP view showed some radiopaque nodules in the lung parenchyma closely related to the pulmonary vasculature (Fig. 1). Transthoracic echocardiogram showed mild left and right ventricular dysfunction (TAPSE: 16 mm; FAC: 33%).

**Fig. 2:**
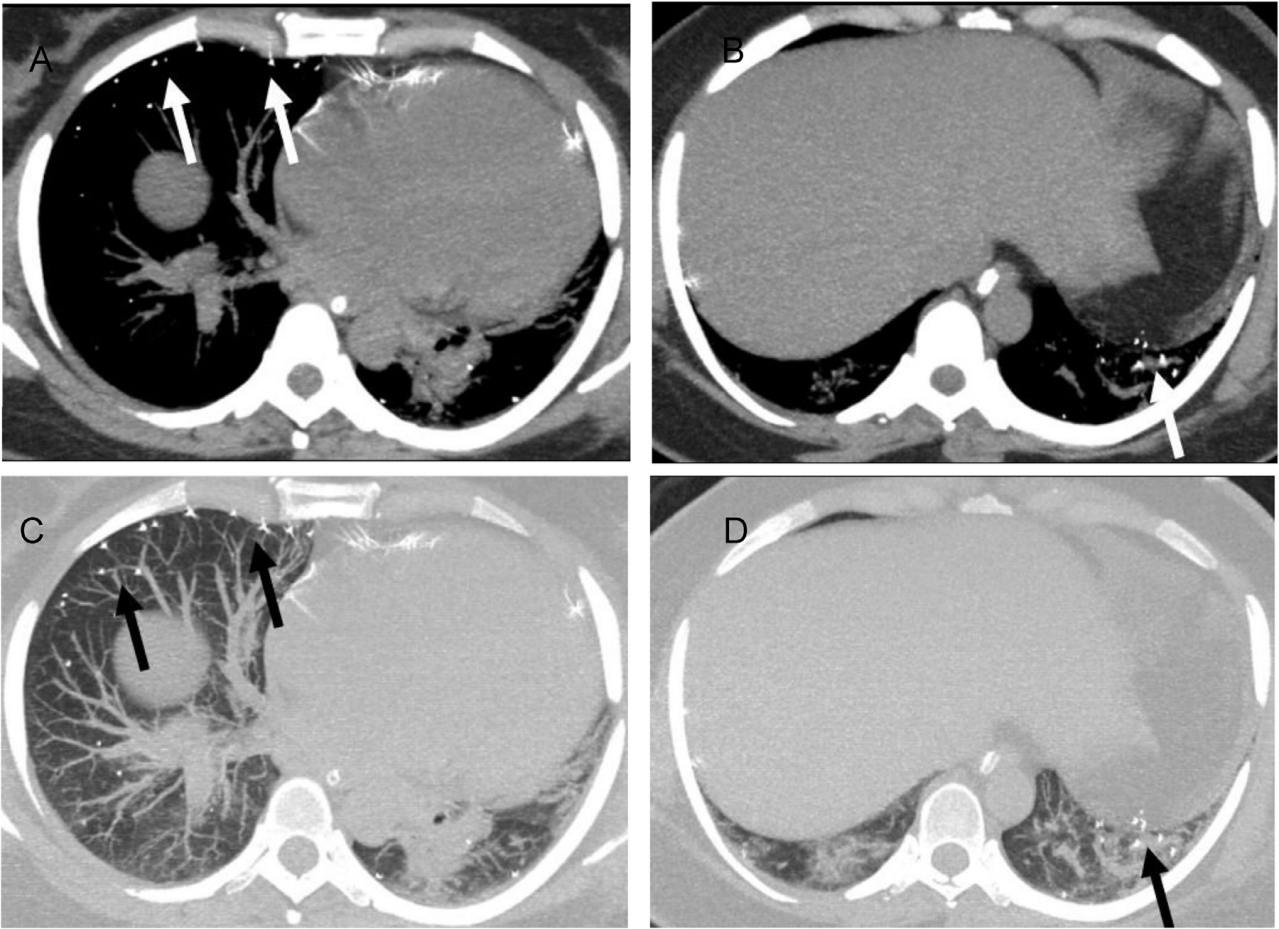
Chest axial CT scan in MIP reconstruction. (A and B) The mediastinal window and (C and D) the lung window shows some hyperdense, rounded, and oval nodules in the lung parenchyma closely related to the pulmonary arteries. There are also similar foci in the right ventricle.

Thoracic chest CT demonstrated pulmonary embolism involving the right lower pulmonary lobe artery (Fig. 2). Systemic thrombolysis with tenecteplase was administered. Un-enhanced CT brain scan revealed ischemic infarcts in the left frontal, parietal, and occipital lobes, as well as the right parietal and occipital lobes, including the postcentral gyrus (Fig. 2). Later, she developed coldness and pallor in the right lower limb with diminished distal pulses. CT angiography revealed thrombosis of the right common femoral artery extending to the superficial and deep femoral arteries, popliteal artery, and infrapopliteal trunks, along with subacute thrombosis of the left popliteal artery, extending to the tibioperoneal trunk and posterior tibial artery (Fig. 2). Emergent thrombectomy was required. Also, deep vein thrombosis, in the left popliteal vein was encountered in a Doppler ultrasound examination (Fig. 2).

**Fig. 3:**
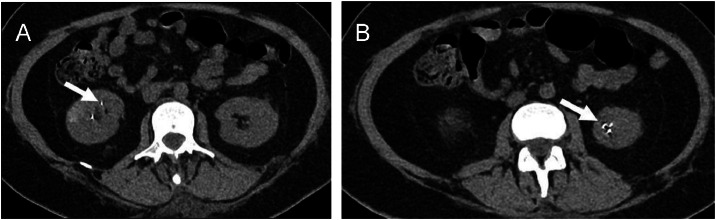
Abdominal axial CT scan in soft tissue window. (A) Unenhanced CT shows hyperdense foci in the inferior renal poles (arrows). (B) Enhanced CT revealed a relation of these foci with the renal arteries and some hypodense cortical wedge-shaped lesions corresponding to renal infarcts.

**Fig. 4:**
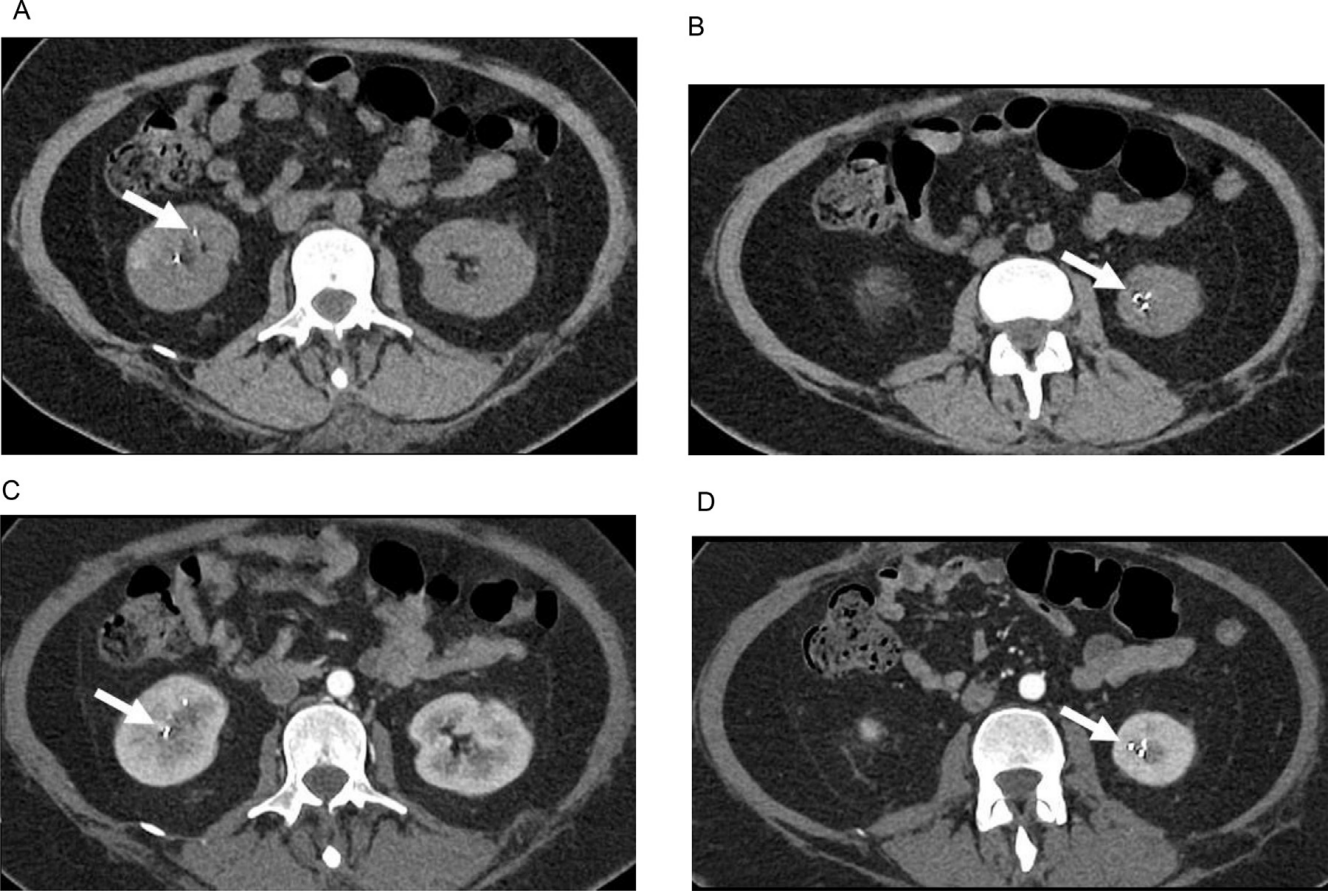
Abdominal axial CT scan in soft tissue window. (A and B) Unenhanced CT shows hyperdense foci in the inferior renal poles (arrows). (C and D) Enhanced CT revealed a relation of these foci with the renal arteries.

Hypercoagulability workup was negative for antinuclear antibodies, anti-beta-2 glycoprotein I IgG/IgM, and anticardiolipin IgG/IgM antibodies. Unenhanced CT scans of the neck, chest, and abdomen demonstrated multiple hyperdense structures within the lungs, cardiac silhouette, liver, kidneys, uterus, and pelvis, suggestive of metallic embolization into vascular structures (Figs. 3 and 4). Blood and urine heavy metal screening showed negative results for arsenic and lead. However, 24-hour urinary mercury levels were 64.03 µg/L (reference <10 µg/L), confirming mercury poisoning, likely via intravenous exposure given the extent and distribution of metallic deposits.

Treatment with oral succimer was initiated at 500 mg every 8 hours for 5 days (not exceeding the maximum dose), followed by 500 mg every 12 hours for 14 days. Intravenous N-acetylcysteine was administered at 150 mg/kg/day for 3 days, then reduced to 50 mg/kg/day for 90 days.

The patient’s clinical condition improved, with successful weaning from vasopressor support and progressive respiratory recovery. She was extubated and entered physical and respiratory rehabilitation. Cognitive and motor neurological sequelae persisted. Despite the thorough history-taking, no definitive source or confirmation of intravenous mercury administration was identified.

Informed consent was obtained from the patient's family for the publication of this case report.

## Discussion

The clinical spectrum of mercury intoxication varies depending on dose, duration, and route of exposure [1]. Acute manifestations may include fever, headache, and myalgia [9], often attributed to corrosive effects. Oral exposure is most common, resulting in gastrointestinal symptoms such as nausea, vomiting, and abdominal pain; severe cases may present with GI bleeding. Inhalation of mercury vapor can lead to bronchitis, pneumonitis, and even acute respiratory distress syndrome (ARDS) [1]. Intravenous exposure may result in embolization to various organs, especially pulmonary vasculature [5], as observed in this patient.

Chronic mercury exposure mainly affects the central nervous system, with neuropsychiatric manifestations including mood disturbances, cognitive impairment, tremors, and ataxia [10]. Renal involvement is the second most frequent consequence, with high urinary mercury levels indicating significant renal accumulation, causing tubular and glomerular damage. Proteinuria and nephrotic syndrome are common presentations [11]. Histological findings often mimic membranous nephropathy, leading to misdiagnosis as primary glomerulopathy [12], as seen in this case.

Pulmonary embolism [5,13] and cerebrovascular accidents [14] secondary to mercury exposure have been documented. Multiple mechanisms contribute to mercury-induced hypercoagulability [15], including oxidative stress, mitochondrial dysfunction, reduced selenium-dependent antioxidant capacity [16], enhanced platelet activation, elevated thrombin and factor VIII levels, and reduced protein C activity [17]. Mercury also promotes vascular smooth muscle proliferation and endothelial inflammation via proinflammatory cytokines such as interleukin-6 and TNF-α, contributing to atherosclerosis, systemic hypertension, and cerebrovascular disease [15]. In this case, multiple arterial and venous thrombotic events were present, with no other identifiable cause of hypercoagulability. Metallic embolization in multiple organs raised clinical suspicion, facilitating diagnosis.

Treatment depends on exposure type and chemical form. Acute poisoning requires chelation with agents like D-penicillamine or DMPS. Chronic exposure is better managed with oral succimer (DMSA), which enhances urinary excretion with fewer side effects. The role of dialysis is controversial due to mercury’s high protein-binding affinity [18]. This patient underwent a 19-day course of succimer with partial clinical improvement. However, due to the uncertain timeline of exposure, treatment efficacy and prognosis remain unclear.

## Conclusions

Mercury poisoning remains underrecognized in our region. Its multisystemic and variable presentation often mimics more common disorders. A thorough clinical history and detailed inquiry into potential exposure sources are critical for early diagnosis. Imaging findings of metallic emboli support the diagnosis and prompt heavy metal screening. Chronic exposure, compounded by slow elimination, predisposes to long-term complications and sequelae.

## Patient consent

Informed consent was obtained from the patient’s legal guardian for publication of this case report "Mercury Poisoning in a Patient with Multiple Arterial and Venous Thrombotic Events: A Case Report" and any accompanying images. The authors confirm that all efforts have been made to protect the patient’s identity and privacy in accordance with the journal’s guidelines.
